# Application of Ultrasound for the Modification of Techno-Functional and Physicochemical Properties in Cereal and Pseudocereal Protein Isolates: A Systematic Review

**DOI:** 10.3390/foods15193498

**Published:** 2026-09-29

**Authors:** Guillermo Segundo Miñan-Olivos, Gonzalo Gastón Palazolo, Julio Vidaurre-Ruiz, Darío Marcelino Cabezas

**Affiliations:** 1 Programa de Doctorado en Ingeniería Agroindustrial Mención Transformación Avanzada de Granos y Tubérculos Andinos, Universidad Nacional del Santa, Nuevo Chimbote 02712, Peru; 2024818003@uns.edu.pe; 2 Laboratorio de Investigación de Funcionalidad y Tecnología de Alimentos (LIFTA), Departamento de Ciencia y Tecnología, Universidad Nacional de Quilmes, Buenos Aires B1876BXD, Argentina; gonzalo.palazolo@unq.edu.ar; 3 Centro de Investigación e Innovación en Productos Derivados de Cultivos Andinos CIINCA, Universidad Nacional Agraria La Molina, Lima 15024, Peru; vidaurrejm@lamolina.edu.pe; 4 Consejo Nacional de Investigaciones Científicas y Técnicas (CONICET), Buenos Aires B1876BXD, Argentina

**Keywords:** acoustic cavitation, structural modification, protein functionality, power ultrasound

## Abstract

Cereal and pseudocereal protein isolates are increasingly investigated as sustainable food ingredients, although their limited solubility, interfacial activity, foaming performance, and hydration properties can restrict their technological application. This systematic review evaluated the effects of ultrasound on the physicochemical and techno-functional properties of protein isolates from cereals and pseudocereals. The review followed PRISMA guidelines and included 26 experimental studies identified from Scopus, Web of Science, Wiley, and Consensus. Owing to substantial heterogeneity in protein sources, sonication conditions, analytical methods, and outcome metrics, the evidence was synthesized narratively rather than by meta-analysis. Overall, ultrasound promoted aggregate disruption, particle-size reduction, partial unfolding, and greater exposure of hydrophilic and hydrophobic groups, which generally improved solubility, emulsifying activity and stability, foaming capacity and stability, water- and oil-holding capacities, and, in some studies, in vitro digestibility. The magnitude and direction of these effects depended on botanical origin and protein composition, as well as on pH, treatment time, acoustic power, and the sequence of combined processes. Treatments integrating ultrasound with pH shifting, heat, glycation, enzymatic modification, or high hydrostatic pressure often produced greater functional improvements than ultrasound alone. Ultrasound is therefore a promising approach for tailoring cereal and pseudocereal protein functionality, although standardized acoustic reporting and scale-up studies remain necessary.

## 1. Introduction

The world’s population is experiencing a considerable increase, together with rapid urbanization and a marked change in consumer eating habits; as a result, in recent years, there has been a growing search for new protein sources with a low environmental impact that can simultaneously ensure sustainable production [1]. In view of this reality, proteins are considered indispensable macronutrients in human nutrition, as they support and regulate nearly all physiological processes. It should also be noted that, historically, animal-derived proteins have been regarded as the main dietary source because of their complete amino acid profiles and high bioavailability; however, the excessive consumption of meat and meat products (rich in saturated fats and cholesterol) has been associated with health risks such as cardiovascular disease, hypertension, and colorectal cancer [2]. Consequently, plant-based proteins have received increasing attention as alternative protein sources because of their nutritional potential, generally lower environmental impact, and compatibility with more sustainable food systems [3,4].

The growing interest of the food industry in using proteins is due not only to their nutritional potential but also to their techno-functional properties, which are considered for the application of novel, high-quality food products [5,6]. Properties such as foam formation, emulsification, and solubility stand out for the improvements they confer on diverse food formulations; however, these depend directly on the chemical composition and molecular structure of the proteins, which requires a meticulous examination of how they behave under various industrial processing conditions. Consequently, the food industry requires viable and efficient approaches to the use of proteins; therefore, there is a growing scientific interest in improving the production of protein concentrates and isolates, or in identifying new sustainable sources.

Specifically, a protein isolate is a purified fraction from plant or animal sources that typically contains more than 90% protein on a dry basis [7]. However, some studies and commercial formulations employ the term isolate with reference to the method of production, omitting the exact protein content, even when it falls slightly below the commonly accepted threshold, namely, between 80% and 90% [8]. By contrast, protein concentrates generally exhibit lower protein purity than isolates and retain larger proportions of carbohydrates and other non-protein constituents because they undergo less intensive fractionation and purification processes; nevertheless, their techno-functional performance cannot be inferred exclusively from protein purity, since botanical origin, extraction procedure, residual composition, and the extent of protein denaturation jointly determine properties such as solubility, emulsification, foaming, water and oil retention, and gel formation [9,10].

Regarding processing, conventional extraction methods such as alkaline solubilization usually recover only part of the protein present in a plant matrix and leave behind residues with a considerable amount still unused, and on top of that, some common operations like heating, homogenizing, or drying can denature proteins partially or even completely [11], so to get around these drawbacks, different physical and enzymatic pretreatments have been explored to release more protein while keeping its structure, with ultrasound standing out because it is efficient and can drive controlled changes in proteins without adding complexity to the process [12,13].

Even so, the success of ultrasound is not automatic, since its effect depends on how frequency, intensity, treatment time, and temperature are set, and these need to be tuned to the specific matrix and the goal of the process [14]; when applied to plant materials, the technique generates cavitation, shear, and turbulence that make solubilization easier and tend to reduce particle size, while also reshaping secondary, tertiary, and even quaternary structures [15].

Across studies on legumes and oilseeds, a recurring picture emerges in which solubility increases as macromolecular aggregates break apart and hydrophilic groups become more exposed [16,17,18,19,20], particle size goes down and this favors foaming and gelation because there is more surface available for protein–protein and protein–liquid interactions [21,22], the net charge and surface hydrophobicity shift in ways that directly influence emulsification and foam formation [20,23], and, finally, oil-holding capacity and emulsion stability often improve after ultrasonic pretreatment, a behavior that has been linked to the development of α-helix and β-turn features in the protein structure [24].

Although numerous reviews have examined the effects of ultrasound on proteins derived from legumes and oilseeds, evidence specifically focused on cereal and pseudocereal protein isolates remains scarce and fragmented. Their predominant protein fractions—prolamins and glutelins in cereals and albumins and globulins in pseudocereals—differ in their structural and compositional characteristics [25], which may lead to distinct responses to acoustic cavitation and limit the direct extrapolation of findings obtained from other botanical matrices. These compositional differences may influence protein hydration, intermolecular association, and the accessibility of hydrophilic and hydrophobic regions, thereby modulating the extent to which ultrasound promotes aggregate disruption, conformational rearrangement, or subsequent reassociation. Therefore, this systematic review aimed to critically synthesize the experimental evidence concerning the effects of ultrasound, applied alone or in combination with other processing treatments, on the physicochemical and techno-functional properties of cereal and pseudocereal protein isolates. Overall, the available evidence indicates that ultrasound generally promotes aggregate disruption, particle-size reduction, partial protein unfolding, and improvements in solubility, interfacial functionality, hydration properties, and digestibility; however, the magnitude and direction of these responses depend on botanical origin, pH, acoustic conditions, and treatment sequence, while inconsistent reporting of processing parameters continues to limit cross-study comparison and industrial scale-up.

## 2. Materials and Methods

The present study employed the PRISMA (Preferred Reporting Items for Systematic Reviews and Meta-Analyses) method to ensure a systematic, comprehensive, and reproducible approach, in contrast to narrative reviews or those lacking a previously established protocol. PRISMA provides a standardized framework for searching, selecting, and analyzing relevant literature in a given field, thereby reducing bias and documenting each stage of the knowledge synthesis process. The literature search was conducted in Scopus, Web of Science, and Wiley, and was last updated on 5 July 2026, including all studies published up to that date.

### 2.1. Search Strategy and Literature Selection

The literature search and study selection were conducted following the PRISMA framework; in this context, the searches were carried out in Scopus, Web of Science, and Wiley.

Table 1 details the search strings employed. Notably, the queries did not include the terms “cereals” or “pseudocereals,” as many studies omit these terms. Consequently, the authors conducted a broad search to identify the raw materials used in each study. In Web of Science, the initial results were overly extensive owing to the range of available categories, so the search was restricted to the Food Science & Technology category. Additionally, the AI-powered academic search engine Consensus was used as a supplementary discovery tool to identify potentially relevant studies that might not have been retrieved through the conventional database searches. A specialized prompt focused on the application of ultrasound to improve the physicochemical and techno-functional properties of cereal and pseudocereal protein isolates was employed. Consensus was not used as a substitute for the bibliographic databases; rather, records identified through this tool were subsequently screened according to the same inclusion and exclusion criteria applied to studies retrieved from Scopus, Web of Science, and Wiley, thereby maintaining consistency throughout the study-selection process.

The PICOS framework (Table 2) guided the review by defining the scope of the evidence concerning the effects of ultrasound on cereal and pseudocereal protein isolates. The population comprised protein isolates and equivalent isolated protein fractions derived from cereals and pseudocereals. The intervention included ultrasound-assisted extraction and post-extraction ultrasonic modification, applied either alone or in combination with other processing treatments, including emulsification, pH shifting, heat treatment, high hydrostatic pressure, glycation, enzymatic hydrolysis, and transglutaminase treatment. Comparators comprised untreated protein isolates, conventional extraction or processing methods, and equivalent treatments performed without ultrasound. The outcomes included physicochemical, techno-functional, structural, and digestibility-related properties. Only experimental studies were considered eligible, allowing the effects attributed to ultrasound to be assessed under controlled processing conditions.

### 2.2. Inclusion and Exclusion Criteria

The inclusion and exclusion criteria were defined before the screening process and applied consistently throughout study selection. We retained only studies that quantified at least one physicochemical or techno-functional property, ensuring conceptual consistency across comparisons. These included investigations involving cereals (wheat, maize, rice, etc.) or pseudocereals (quinoa, amaranth, kañiwa, etc.), and publications in peer-reviewed scientific journals. In parallel, studies were excluded when ultrasound was not employed as the primary treatment or as a clearly defined component of the intervention, when the raw material fell outside the scope of cereals and pseudocereals, when duplicate records were identified, or when the experimental treatment, ultrasound conditions, or relevant outcome data were insufficiently described to permit interpretation and comparison within the review. Regarding the operational definition of a “protein isolate,” studies were considered eligible when the material presented a protein content of at least 80% on a dry basis. In cases where protein purity was not explicitly reported, we evaluated whether the authors identified the material as a protein isolate and described an equivalent isolation procedure involving protein solubilization, separation, and precipitation. In these cases, methodological equivalence was established from the reported preparation procedure, and studies were included only when the resulting material was consistent with the type of protein isolate considered within the scope of this review. Differences in protein purity among the included isolates were considered part of the compositional heterogeneity of the evidence, since techno-functional behavior is also influenced by botanical origin, protein fraction composition, extraction history, residual non-protein components, and structural state; therefore, the ≥80% criterion was used as an operational eligibility threshold rather than as an indication of compositional equivalence among isolates.

### 2.3. Study-Selection Process

**Figure 1:**
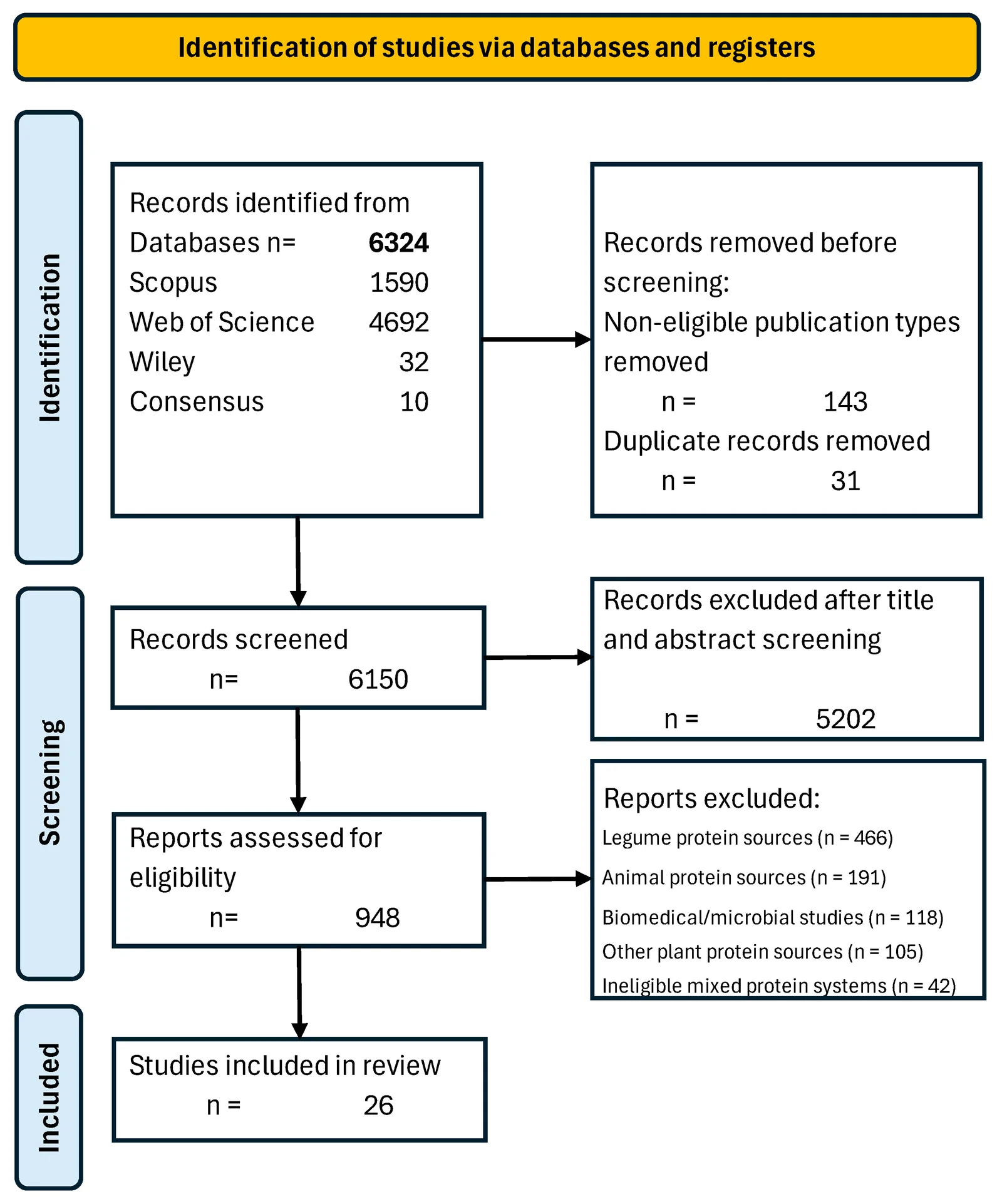
Literature search conducted using the PRISMA methodology.

The records retrieved from Scopus, Web of Science, Wiley, and Consensus were screened by G.S.M.O. and J.V.-R. using the predefined eligibility criteria. Titles and abstracts were assessed first, followed by full-text evaluation of potentially eligible reports. Data extraction was also conducted by G.S.M.O. and J.V.-R. Any discrepancies regarding study eligibility or interpretation of the reported information were resolved through discussion and consensus among the authors. The complete study-selection process is summarized in Figure 1.

### 2.4. PRISMA Methodology and Risk-of-Bias Assessment

The risk-of-bias assessment was grounded in principles of the ROBINS-I (Risk of Bias in Non-randomized Studies of Interventions) framework, adapted to laboratory experimental studies in food science. Specifically, predefined criteria were applied to evaluate potential bias arising from confounding variables; the appropriateness of sample selection and preparation; the classification and description of the intervention with sufficient detail to enable reproduction; deviations from the intended treatment that could alter the effective exposure; data integrity and the handling of missing values; the validity and reliability of measurements of physicochemical and techno-functional outcomes; and selective outcome reporting. These criteria were applied uniformly during full-text review, with the aim of capturing not only the presence of controls and the rigor of experimental design and statistical analysis, but also the internal consistency of reporting and the methodological plausibility of effects attributed to the intervention. The risk-of-bias assessment was conducted independently by at least two authors of the review using the predefined seven-domain framework, and any discrepancies in the assignment of domain-level or overall judgments were resolved through discussion and consensus.

For operational purposes, each of the seven adapted domains was rated as low risk (2 points), moderate risk (1 point), or high risk (0 points), resulting in a maximum total score of 14 points. Studies scoring 12–14 points were classified as having a low overall risk of bias, those scoring 8–11 points as having a moderate risk, and those scoring 0–7 points as having a high risk. These thresholds were selected to distinguish studies with predominantly low-risk domain ratings (12–14 points), studies showing a mixed profile of low- and moderate-risk ratings (8–11 points), and studies in which moderate- or high-risk ratings predominated (0–7 points). This semiquantitative scoring system was developed specifically for the present review to operationalize the adapted domain-based assessment and should not be interpreted as the original ROBINS-I scoring system. Detailed decision rules for each domain, the domain-level scores assigned to the 26 included studies, the corresponding total scores, and the overall risk-of-bias classifications are presented in the Supplementary Materials.

### 2.5. Synthesis Methods

The synthesis was conducted narratively, organizing findings by raw material, ultrasound treatment, and the physicochemical, structural, techno-functional, and digestibility-related properties evaluated. Methodological quality and risk-of-bias judgments are summarized in Table 3, while Table 4 provides a descriptive overview of the properties evaluated in each included study. Data were retained as reported by the original studies, without conversions or imputations. Because the included studies differed substantially in protein sources, experimental conditions, analytical methods, units, and outcome metrics, no meta-analysis or harmonized effect size was calculated; instead, the evidence was integrated through qualitative comparison across comparable groups. The direction and magnitude of the reported effects were evaluated from the original quantitative results and are critically discussed in Section 4.1, Section 4.2, Section 4.3 and Section 4.4. Consequently, PRISMA subitems 13e–13f (statistical heterogeneity and sensitivity analyses) were not applicable.

### 2.6. Registration and Protocol

Registration and protocol (PRISMA Item 24). This review was not registered. We followed an internal, pre-specified, non-public protocol detailing the research question, eligibility criteria, information sources and search strategy, study selection and data extraction procedures, risk-of-bias assessment, and synthesis plan. No post hoc amendments were made.

### 2.7. Availability of Data, Code and Other Materials

The data supporting this systematic review are deposited in Zenodo: https://doi.org/10.5281/zenodo.16928429 (accessed on 18 September 2026). The repository contains the records retrieved from the bibliographic sources searched, together with the data generated during the screening and study-selection process. Access to these data is restricted and will be granted upon request to the corresponding author, who will provide access to interested researchers. The synthesis tables and derived analyses presented in the manuscript are not included in the repository but can also be provided through the same request mechanism.

## 3. Results

Table 3 summarizes the methodological characteristics and overall risk-of-bias assessment of the 26 included studies. Most investigations used factorial or comparative experimental designs, although Box–Behnken, orthogonal, central composite, and response-surface approaches were also applied. Considerable heterogeneity was observed in the reporting and control of ultrasound conditions, including frequency, power, amplitude, treatment time, duty cycle, temperature management, and treatment geometry, which limited direct comparability across studies. It should be noted that the acoustic descriptors reported across studies are not directly interchangeable. Nominal power, actual acoustic power, amplitude, acoustic intensity, and energy density represent different physical or operational quantities and were therefore retained as originally reported. No conversions were performed when the information required for valid normalization was unavailable. Most studies included an untreated protein, a conventional extraction procedure, or an individual treatment as a comparator and analyzed the data using ANOVA followed by post hoc tests such as Tukey, Duncan, or LSD. Based on the adapted domain-level assessment, 17 studies were classified as having a low overall risk of bias and 9 as having a moderate risk, whereas none were classified as high risk. The most frequent methodological limitations were related to incomplete characterization of the ultrasonic intervention, insufficient control or monitoring of treatment deviations, particularly temperature changes during sonication, incomplete reporting of replication or dispersion measures, and, in some studies, limitations in sample or comparator equivalence.

The included studies comprised both ultrasound-assisted extraction and post-extraction ultrasonic modification. In extraction-stage studies, ultrasound could influence protein recovery, purity, and composition, which may in turn affect the physicochemical, structural, and techno-functional properties of the resulting protein isolate. In these cases, extraction-related variables were considered contextual factors rather than primary outcomes of the review. By contrast, post-extraction treatments acted directly on previously isolated protein systems and therefore reflected more specifically the structural and functional modification of the protein fraction. In all cases, the synthesis and discussion focused on the modifications observed in the physicochemical, structural, and techno-functional properties of the resulting protein isolates.

Table 4 provides an overview of the main physicochemical, structural, techno-functional, and digestibility-related properties evaluated across the included studies. Specifically, S represents solubility, EAI/ESI emulsifying activity or stability, FC/FS foaming capacity or stability, WHC/OHC water- and oil-holding capacity, IVPD in vitro protein digestibility, Particle size the assessment of particle-size characteristics, and Str 2°/3° the evaluation of secondary and/or tertiary protein structure. The table is intended to describe the distribution of the outcomes investigated in the available literature rather than the direction or magnitude of the effects produced by ultrasound.

An “x” indicates that the corresponding property was evaluated and reported in the original study. NA indicates that the property was not assessed, NR that the corresponding result was not reported, and NC that the available information did not permit classification within the respective outcome category. These indicators therefore describe only the coverage and availability of the evaluated properties and do not represent the direction or magnitude of the ultrasound-induced effects.

The available evidence was unevenly distributed across protein sources, with quinoa and oat accounting for 16 of the 26 included studies, whereas several other cereal and pseudocereal protein systems were represented by only one or a few studies. Consequently, the overall trends identified from the reviewed evidence may reflect more strongly the behavior of these better-represented protein systems and should not be extrapolated uniformly to underrepresented cereal proteins, particularly those rich in relatively recalcitrant prolamin fractions.

**Figure 2:**
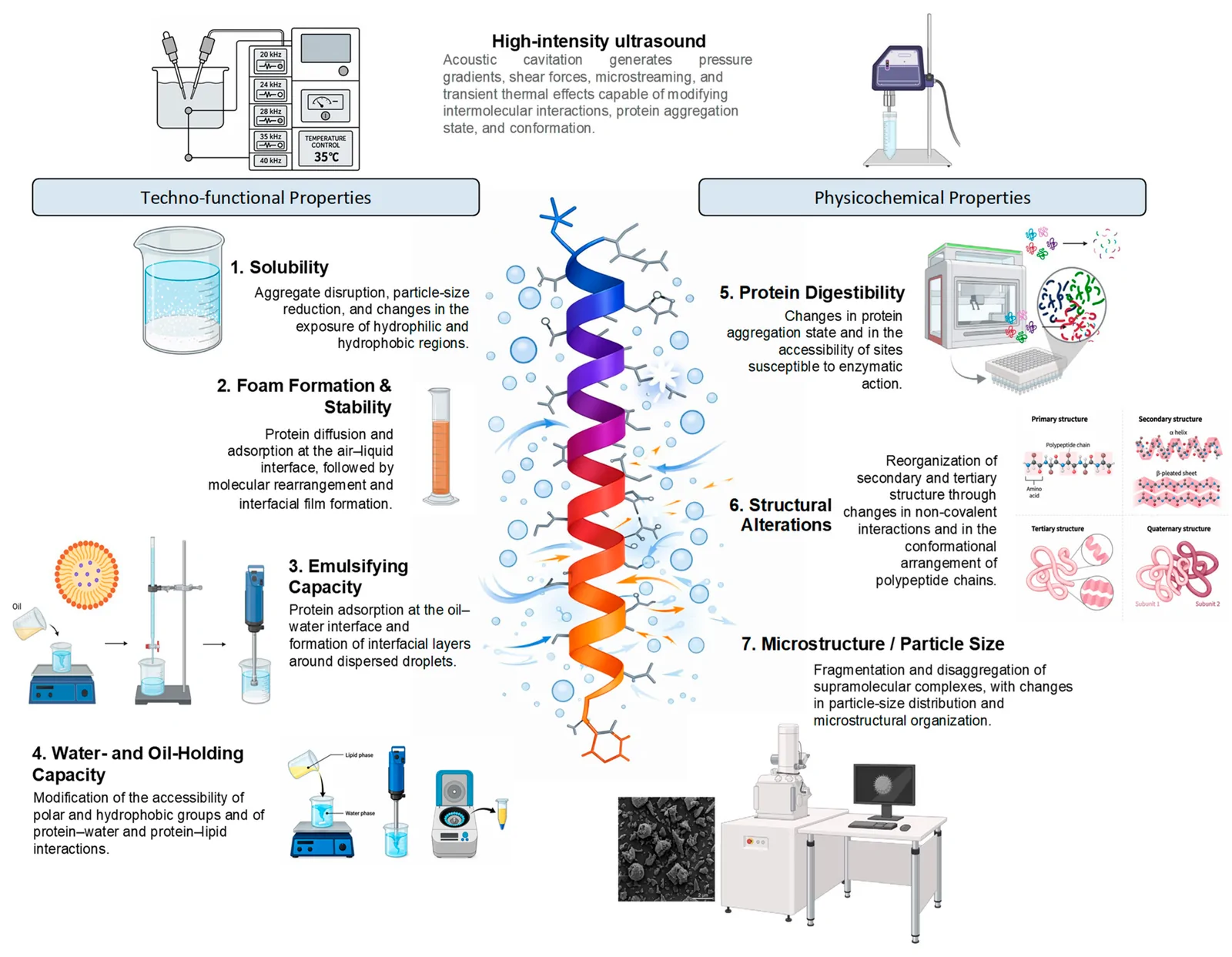
Schematic representation of the main physical and physicochemical phenomena associated with high-intensity ultrasound treatment of cereal and pseudocereal protein isolates. Note: The schematic summarizes commonly reported mechanistic phenomena and should not be interpreted as representing universal directional responses. The nature and extent of individual changes may vary according to protein source, ultrasound conditions, processing sequence, and the structural state of the protein system.

Figure 2 presents a schematic representation of the main physical and physicochemical phenomena associated with the application of high-intensity ultrasound to cereal and pseudocereal protein isolates. The collapse of cavitation-induced microbubbles generates localized pressure gradients, shear forces, microstreaming, and transient thermal effects that can modify intermolecular interactions, protein aggregation state, and molecular conformation. These phenomena are associated with (i) aggregate disruption, particle-size reduction, and changes in the exposure of hydrophilic and hydrophobic regions related to solubility; (ii) protein diffusion, adsorption, and rearrangement at the oil–water interface, which are relevant to emulsifying behavior; (iii) protein diffusion and adsorption at the air–liquid interface and subsequent interfacial film formation, which are associated with foaming properties; (iv) changes in the accessibility of polar and hydrophobic groups and in protein–water and protein–lipid interactions, which influence water- and oil-holding capacities; (v) fragmentation and disaggregation of supramolecular complexes, with consequent changes in particle-size distribution and microstructural organization; (vi) changes in protein aggregation state and in the accessibility of sites susceptible to enzymatic action, which may affect digestibility; and (vii) condition-dependent rearrangements of secondary and tertiary structure resulting from modifications in non-covalent interactions and in the conformational organization of polypeptide chains.

## 4. Discussion

### 4.1. Mechanisms of Solubility Enhancement: Aggregate Disruption and Particle-Size Reduction

Solubility governs the dispersibility and technological performance of protein isolates in aqueous food systems. Across the reviewed studies, ultrasound generally improved this property by disrupting supramolecular aggregates and increasing the accessibility of protein regions capable of interacting with water [17]. Nevertheless, the magnitude of the response depended on the initial organization of the protein matrix and on whether the applied acoustic treatment remained below the point at which reassociation or excessive denaturation became dominant.

This non-linear behavior was evident in amaranth and quinoa proteins. Tomé and García [34] showed that solubility depended jointly on sonication amplitude and treatment time. Similarly, Li et al. [39] reported that moderate power enhanced the solubility of quinoa protein hydrolysates across a broad pH range but that the highest power reduced the benefit. Mir et al. [35] likewise observed progressive fragmentation and greater surface irregularity up to an intermediate treatment time, followed by a partial loss of functionality after prolonged sonication. Taken together, these studies indicate that acoustic energy must be sufficient to overcome the interactions maintaining protein aggregates, but not so intense that newly exposed hydrophobic regions favor secondary association.

The pH environment and the sequence of combined operations further determined the solubilization response. Igartúa et al. [28] obtained the greatest solubility when ultrasound was applied after pH shifting and heating, indicating that proteins previously modified by alkaline and thermal treatment were more susceptible to subsequent acoustic disruption. Hegde et al. [26] and Luo et al. [31] also found greater solubility when ultrasound was integrated with high-pressure treatments, although the response remained limited near the isoelectric region. Similar pH dependence was reported for barley [37], while ultrasound-assisted glycation increased buckwheat protein solubility beyond that achieved by conventional heating [40]. Comparable improvements reported for other ultrasound-assisted chemical and enzymatic modification routes [52,53] support the view that complementary treatments are most effective when they first weaken the interactions that restrict protein dispersion.

Particle-size measurements provided direct evidence of aggregate disruption across cereal matrices. Dual-frequency ultrasound reduced the mean diameter of rice-residue protein isolates from approximately 460 to 220 nm [36]. Likewise, ultrasound combined with pH shifting decreased the average size of barley protein isolate from 27.5 to 4.9 µm and reduced sedimentation [37]. In the soy-protein-wheat-gluten system evaluated by Qin et al. [41], both surface- and volume-weighted diameters decreased by more than half. These results show that particle-size reduction is a recurrent physical consequence of sonication, although its functional relevance depends on whether the resulting particles remain dispersed rather than reassociating after treatment.

Evidence from oat protein isolates confirmed the same relationship. Li and Xiong [45] found that high-intensity ultrasound reduced particle size by up to 37% and increased solubility by up to 48% outside the isoelectric region. These changes occurred without alterations in the polypeptide profile or in the disulfide bonds connecting the α- and β-subunits. Li et al. [47] subsequently showed that thermosonication followed by high-pressure homogenization progressively reduced particle size up to an intermediate pressure, after which additional reductions became comparatively small. The treatment also increased solubility at neutral pH. A more moderate response was reported by Li et al. [46], who observed a decrease in mean diameter from 381.1 to 253.1 nm after HIU alone. Thus, ultrasound modified oat protein predominantly through aggregate disruption rather than extensive cleavage of the primary protein structure.

Quinoa protein systems exhibited some of the largest reductions in particle size. In particles used to stabilize Pickering emulsions, sonication decreased the diameter from approximately 401 to 207 nm [38]. Cen et al. [42] similarly reported a reduction in the Z-average diameter from 463.87 nm to 123.84–143.47 nm, together with a narrower particle-size distribution. Zuo et al. [51] reported a decrease from 796.13 ± 115.34 nm in untreated QPI to 249.23 ± 6.25, 207.10 ± 15.68, and 173.40 ± 6.98 nm at progressively higher acoustic-energy densities. The largest proportional reduction occurred during the first increase in energy input, while subsequent increments produced smaller additional changes. Yang et al. [50] similarly found that ultrasonication generated a greater size reduction than thermal-acid or enzymatic hydrolysis and increased solubility from 53.3% to 59.4%.

The effect was intensified when the protein had undergone prior conformational modification. Wang et al. [49] reported that pH shifting followed by sonication reduced particle size more strongly and increased solubility to approximately 90%, outperforming the individual treatments. Chen et al. [43] also obtained QPI-gum Arabic conjugates with greater solubility through ultrasound-assisted wet heating than through conventional wet heating. These findings demonstrate that ultrasound can act both as a direct disaggregation treatment and as an intensifying step within combined processes. However, the observed improvement should be attributed to the complete treatment sequence rather than to sonication alone.

### 4.2. Interfacial Functionality: Foaming, Emulsification, Wettability, and Surface Charge

Foaming and emulsifying performance depend on the ability of proteins to reach an interface, reorganize, and form a sufficiently cohesive layer around air bubbles or oil droplets. Reviews of ultrasound-generated protein emulsions indicate that sonication can favor these processes by modifying molecular flexibility and interfacial accessibility [54], and comparable responses have been observed in other plant protein isolates subjected to ultrasound-assisted modification [55]. Within cereal and pseudocereal systems, however, the interfacial response was not uniform and depended strongly on pH, botanical origin, protein concentration, and the severity of treatment.

For foaming properties, the most favorable responses were generally obtained when ultrasound increased protein dispersion without causing extensive reassociation. In pearl-millet protein, the combined ultrasound-high-pressure treatment increased foaming capacity from 18 ± 2% to 44 ± 1% and foaming stability from 38 ± 1% to 54 ± 1% [26]. Wheat-germ protein showed its strongest foaming response above neutral pH and its weakest response near the isoelectric region [30], confirming that limited solubility restricts rapid adsorption at the air-water interface. Omura et al. [33] further demonstrated that botanical source can override a common processing effect, because soy protein showed a pronounced increase in foaming capacity whereas rice protein improved only under a restricted set of conditions.

When interpreting emulsification-related outcomes, it is necessary to distinguish between two experimental approaches. In some studies, ultrasound was applied to the protein system before emulsion preparation, whereas in others sonication was applied during emulsion formation. In the former case, the emulsion mainly serves as a functional test of a previously modified protein, whereas in the latter, protein reorganization, droplet disruption, and interfacial adsorption occur concurrently.

A comparable matrix dependence was observed for emulsification. Yang and Cheng [27] produced highly structured quinoa protein emulsions at a lower oil fraction than that required with a conventional rotor–stator device. This result indicates that ultrasound promoted both emulsion formation and interfacial organization of the protein system. Similarly, He et al. [29] evaluated quinoa protein-EGCG hybrid particles in Pickering emulsions prepared with ultrasound. The reduction in droplet growth and the greater resistance to physical destabilization reflected the combined effects of the protein-containing particles, droplet disruption, and interfacial organization during sonication. In rice protein, by contrast, ultrasound was applied to the protein system before the subsequent evaluation of emulsifying properties. Only selected treatments increased the emulsifying activity index, whereas emulsion stability improved more consistently [33]. Accordingly, emulsification-related outcomes should be interpreted according to the stage at which ultrasound was applied, since prior protein modification and concurrent ultrasonic emulsification represent mechanistically different experimental conditions.

The order and intensity of processing also affected interfacial functionality. Hegde et al. [26] found that reversing the sequence of ultrasound and high-pressure treatment produced the highest emulsifying activity in pearl-millet protein. This finding indicates that the structural state generated by the first operation conditioned the effect of the subsequent treatment. In amaranth protein, emulsifying activity increased across several sonication conditions, whereas the most intense treatment decreased emulsifying stability [34]. Mir et al. [35] reported a similar optimum in quinoa protein, with activity and stability increasing up to an intermediate treatment and then declining. Therefore, greater acoustic exposure does not necessarily generate a stronger interfacial film, because excessive structural disruption may favor reassociation or reduce the capacity of proteins to reorganize at the interface.

Ultrasound-assisted conjugation produced additional interfacial benefits. Xue et al. [40] showed that ultrasound-assisted glycation increased both the emulsifying activity and stability of buckwheat protein–dextran conjugates beyond the values obtained by conventional heating. Chen et al. [43] likewise found that ultrasound-assisted QPI-gum Arabic conjugates exhibited higher emulsifying activity than conventionally heated conjugates for oregano, Litsea cubeba, and cinnamon essential oils. Because the magnitude of the response varied with the oil phase, the functional advantage cannot be assigned exclusively to the degree of conjugation; it also reflects the compatibility between the modified interfacial material and the dispersed lipid.

By contrast, in other studies, the protein isolate or protein particles were first modified by ultrasound and subsequently evaluated as emulsion stabilizers. Under this experimental approach, the emulsification step served primarily as a test of the functionality of the previously modified protein system. Qin, Luo, and Peng [38] found that sonicated quinoa protein nanoparticles decreased droplet size and creaming, although the final stability continued to depend on protein concentration and dispersed-phase fraction. Cen et al. [42] observed that sonication increased the water contact angle from 53.69 ± 0.84° to 68.83 ± 3.32°, shifting the particles toward a more balanced wettability that favored their retention at the oil–water interface. These results show that the stabilizing action of ultrasound-treated particles arose from the combined contribution of particle organization, wettability, and interfacial packing rather than from size reduction alone.

More recent studies further clarified the role of surface properties. Li et al. [47] found that the oat protein treatment producing the best emulsion viscosity and physical stability also improved emulsifying activity, although the highest homogenization pressures provided little additional benefit. Wang et al. [49] reported the best overall foaming and emulsifying performance when pH shifting was combined with sonication. Under these conditions, foaming capacity reached approximately 170%, foaming stability approximately 45%, and EAI approximately 80 m^2^ g^−1^. Nevertheless, a heat-containing treatment achieved a comparable ESI, indicating that ultrasound was not superior for every interfacial outcome. Zuo et al. [51] observed only small changes in the initial ζ-potential of QPI dispersions, which remained approximately −30 to −32 mV. However, after interfacial adsorption, sonicated particles retained a substantially more negative charge than untreated QPI. Consequently, enhanced emulsification cannot be explained solely by an increase in initial net charge and should instead be interpreted as the combined result of structural modification and improved preservation of particle properties at the interface.

### 4.3. Network Refinement and Enhancement of Water- and Oil-Holding Capacities

Water- and oil-holding capacities describe the ability of protein ingredients to associate with polar and non-polar phases, respectively, and are relevant to texture, yield, and stability in formulated foods [56]. Ultrasound can alter both properties by changing the accessibility of hydrophilic and hydrophobic regions [57], but the reviewed evidence indicates that hydration and lipid retention do not necessarily increase simultaneously. Moreover, the behavior of a protein powder or dispersion must be distinguished from water immobilization within a heat-set or enzyme-induced gel network.

Among cereal proteins, Hegde et al. [26] reported that the combined treatment increased the water-holding capacity of pearl-millet protein from 2.42 ± 0.07 to 3.2 ± 0.1 g g^−1^. Oil-holding capacity also increased, from 2.9 ± 0.1 to 3.94 ± 0.09 g g^−1^. Xie et al. [30] found lower water absorption but higher oil retention in wheat-germ protein than in the soy comparator, indicating that botanical composition conditioned the balance between hydration and lipid association. Omura et al. [33] likewise observed strong but highly condition-dependent responses in rice and soy proteins. Therefore, large increases reported for a particular source should not be generalized without considering protein composition, concentration, and the measurement procedure used.

Quinoa studies confirmed the non-linear nature of these properties. Mir et al. [35] observed that both water- and oil-holding capacities increased up to an intermediate sonication time and declined moderately after prolonged treatment. By contrast, Li et al. [39] found that ultrasound pretreatment decreased the water-holding capacity of quinoa protein hydrolysates while increasing oil absorption, particularly at the highest power. This divergence demonstrates that the exposure of hydrophobic regions can improve lipid association even when the capacity to retain water decreases, and it cautions against interpreting all functional changes as a uniform improvement.

Water retention in gelled systems depended more directly on the organization of the final protein network. Li et al. [46] found that HIU alone reduced cooking loss in oat protein gels by up to 76% relative to untreated gels. Proton nuclear magnetic resonance measurements showed no bulk or highly mobile water in the HIU-modified gels, together with shorter relaxation times for immobilized water. These findings indicate that the modified network restricted water mobility more effectively during heating. The result is important because it demonstrates that macroscopic water retention is determined not only by molecular hydration but also by the continuity and pore structure of the gel formed after sonication.

A comparable response was observed in the soy–protein–wheat–gluten system evaluated by Qin et al. [41]. Ultrasonic pretreatment facilitated subsequent network formation and increased water retention to 85.47 ± 0.70%, compared with approximately 60–70% in untreated gels. In both oat and wheat-gluten-containing systems, ultrasound acted primarily as a structural pretreatment that enabled the proteins to reorganize into a denser matrix during gelation. Accordingly, the effect of sonication on water retention should be assessed in relation to the final network formed, rather than inferred only from changes measured in the untreated dispersion.

### 4.4. Conformational Unfolding and Enhancement of Protein Digestibility

Ultrasound modified secondary and tertiary protein organization, but the direction of the response was not uniform across matrices. In buckwheat protein, ultrasound-assisted glycation markedly reduced α-helix content and increased less-ordered structures [40], while quinoa studies reported partial unfolding after sonication or combined pressure treatment [31,39]. Qin et al. [41] similarly observed decreases in α-helix and β-turn structures together with increases in β-sheet and random-coil contents in a soy–protein–wheat–gluten mixture. These findings indicate that cavitation commonly disrupts stabilizing interactions, although the structure measured after treatment also reflects subsequent molecular reassociation.

Changes in tertiary structure were not always accompanied by a shift in the fluorescence maximum. Mir et al. [35] found that the maximum emission wavelength of quinoa protein remained at 348 nm after sonication, while fluorescence intensity decreased. This pattern indicates modification of the local environment and accessibility of aromatic residues without evidence of a pronounced displacement in the emission maximum. Therefore, fluorescence intensity and wavelength should be interpreted together rather than using either measurement alone as proof of complete unfolding.

The newer studies illustrate why conformational change cannot be described as a simple progression from ordered to disordered structures. Rafique et al. [48] found that the secondary-structure profile of oat protein changed only moderately between the intermediate and highest ultrasound powers. This result indicates that structural modification was not proportional to power input. Zuo et al. [51] observed a decrease in random-coil content from 28.15 ± 1.06% to 25.10 ± 0.28%, together with an increase in α-helix content from 5.65 ± 0.07% to 6.80 ± 0.28%. These changes are consistent with reassociation into a more ordered particle structure. Conversely, Wang et al. [49] reported greater random-coil content and lower proportions of α-helix, β-sheet, and β-turn structures when sonication followed pH shifting. These opposite trends show that the final conformation depends on the structural state before sonication and on the opportunity for proteins to reassociate after acoustic disruption.

The nutritional consequence of these modifications was evaluated in comparatively few studies. Ultrasound-induced disaggregation can increase enzyme accessibility to peptide bonds [58], but this effect is limited when treatment generates compact aggregates or when subsequent processing reduces protease access. Hegde et al. [26] reported an increase in in vitro digestibility from 72 ± 2% to 83 ± 2% after the combined ultrasound-high-pressure treatment. Jin et al. [32] similarly increased buckwheat protein digestibility from 41 ± 2% to a maximum of 58 ± 5% at an intermediate treatment, whereas longer exposure or greater amplitude reduced the improvement. The existence of this optimum supports the interpretation that moderate structural opening promotes hydrolysis, while excessive treatment can partially reverse the benefit.

The sequence of processing must also be considered when a study reports reduced digestibility. He et al. [44] showed that ultrasound-assisted alkaline extraction increased quinoa protein recovery and purity while reducing the time and temperature required for extraction. However, the lower degree of hydrolysis observed in some samples occurred only after the recovered isolate was subjected to additional heating. The strongest reduction was observed after the most severe thermal treatment. Consequently, this study does not demonstrate a detrimental effect of ultrasound-assisted extraction on proteolysis. Instead, it shows that digestibility reflects the complete thermal and structural history of the protein after sonication.

### 4.5. A Critical Evaluation from the Collected Evidence: Methodological Gaps and Future Perspectives

The approach adopted, based on the PRISMA guideline, was a major strength, providing a standardized framework that ensures transparency in the search, selection, and synthesis processes, making each stage visible and auditable. This represents a clear advantage over narrative reviews, which are frequently used to map the state of the art. However, narrative approaches may involve several limitations, including a higher risk of selection bias due to non-exhaustive or non-reproducible searches and the absence of explicit analytical criteria such as PICOS. They may also lack formal assessment of risk of bias and evidence quality, thereby limiting the evaluation of confidence in the findings and the management of heterogeneity across critical variables. Precisely for these reasons, the PRISMA structured reporting scheme adds significant value in experimental domains as complex as that addressed in the present study.

When a literature review is carried out in a systematic way, it is not just about collecting papers, because without a shared vocabulary the search can easily miss important studies, especially in food science. In the present study, basic descriptors such as “cereals” and “pseudocereals” were frequently absent from the metadata used to guide the searches. This reflects the heterogeneous terminology employed across the literature and indicates that conventional search strategies may overlook relevant studies, thereby providing an incomplete representation of the field. For this reason, carefully planned conventional searches were complemented with AI-assisted queries to broaden retrieval and identify studies that might otherwise have been missed [59,60].

Similarly, the studies included in this review were heterogeneous in their experimental execution, particularly with respect to ultrasound power, frequency, treatment duration, and device configuration. This variability limited the possibility of calculating a common effect size and therefore precluded an appropriate conventional meta-analysis. Nevertheless, repeated patterns were observed across the reported results. Rather than considering this variability solely as a limitation [61], it may also reflect a field that is still exploring the relevant processing conditions and clarifying how ultrasound modifies cereal and pseudocereal proteins.

The analysis of the available literature revealed a limited number of studies specifically focused on cereal and pseudocereal protein isolates, particularly when compared with the larger body of research on proteins from legumes and oilseeds. This difference reflects the comparatively emerging nature of research on these protein sources and the increasing attention directed toward underutilized grains with nutritional and technological potential. However, the limited evidence base should not be interpreted solely as a methodological weakness of the present review. Rather, it identifies a knowledge gap that supports the need for additional studies using comparable experimental designs, harmonized outcome metrics, and more comprehensive reporting of acoustic parameters and processing-medium conditions.

The influence of protein source also warrants further comparative investigation. Pseudocereal proteins exhibit particular compositional and techno-functional characteristics that distinguish them from conventional cereal proteins and may influence their response to processing technologies [25]. Previous comparative evidence further indicates that proteins from different botanical origins may exhibit distinct physicochemical and functional responses to ultrasound treatment [14]. More recently, Riquelme et al. [62] observed pronounced structural unfolding and a reduction in aggregate size in pea and lupin proteins, whereas rice protein exhibited comparatively limited structural and microstructural changes under the same sonication conditions. These findings suggest that intrinsic protein composition, aggregation state, and intermolecular interactions may modulate susceptibility to acoustic cavitation. However, direct studies comparing cereal and pseudocereal protein isolates under standardized ultrasound conditions are still lacking; therefore, controlled head-to-head experiments are required before systematic differences between these botanical groups can be established.

It is essential to emphasize that the present review identifies important gaps in the analysis of ultrasound for the processing of protein isolates, which is why further advances remain necessary. In this field, three fundamental strategic priorities can be delineated to enable the industrial maturation of this promising technology. The first priority consists of a comprehensive techno-economic and energy evaluation that quantifies investment and operating costs, energy-conversion efficiency, maintenance requirements, and electrical consumption of the required equipment. Such evaluations should also report specific energy, effective acoustic power, energy density, and functional yield per unit of consumed energy. The second strategic priority concerns technological transfer and industrial scale-up. Progress in this area will require multipoint sonotrodes and acoustic reactors equipped with closed-loop control systems (PLC) to regulate power or amplitude, pressure, temperature, and flow conditions. Reactor design should also consider geometric attenuation, cavitation distribution, and viscous dissipation to improve reproducibility, process uniformity, and operational safety [63,64]. The third strategic priority involves comprehensive environmental validation using tools such as life cycle assessment (LCA). These studies should quantify carbon and water footprints, waste flows, and potential effects on sensory quality and product shelf life, while enabling comparisons with alternative processing technologies [65].

## 5. Conclusions

The present study indicates that ultrasound constitutes a promising technology for modifying protein isolates from cereals and pseudocereals, since acoustic cavitation induces structural changes that directly affect their physicochemical and techno-functional behavior. Across the studies analyzed, ultrasound was associated with the partial unfolding of polypeptide chains, the disruption of aggregates, the reduction in particle size, and a greater exposure of hydrophilic and hydrophobic groups, thereby favoring protein reactivity in food systems.

These structural modifications were reflected in improvements in key techno-functional properties, particularly in solubility, emulsifying capacity, foaming behavior, water- and oil-holding capacities and, in some cases, digestibility. However, the magnitude and direction of these effects depended to a great extent on both intrinsic factors and processing variables, among them botanical origin, pH conditions, treatment time, frequency, and acoustic power, which confirms that the response to ultrasound is polydependent and cannot be generalized to all protein systems.

The evidence also indicates that ultrasound can be particularly effective when applied in combination with complementary strategies such as pH shifting, enzymatic hydrolysis, glycation, or high hydrostatic pressure, since these combined treatments frequently produced greater functional improvements than ultrasound applied in isolation. Nevertheless, despite the favorable trends identified, the high heterogeneity of experimental conditions and the limited standardization in the reporting of acoustic parameters impose a restriction on direct comparability among studies and make it difficult to establish standardized processing conditions.

In this context, future research should prioritize the standardization of methodological criteria, particularly with regard to effective acoustic power, energy density, and treatment conditions; at the same time, progress should be made toward techno-economic evaluation and industrial scale-up. Such progress will be essential to determine the reproducibility, feasibility, and practical relevance of ultrasound-assisted modification of protein isolates from cereals and pseudocereals in the development of sustainable, functional, and high-value food products.

## Figures and Tables

**Table 1:** Search strings and prompts used to retrieve research articles.

Database/Search Engine	Search String/Prompt
Scopus	(TITLE-ABS-KEY (ultrasound) OR TITLE-ABS-KEY (ultrasonication) OR TITLE-ABS-KEY (ultrasonic) OR TITLE-ABS-KEY (sonication) AND TITLE-ABS-KEY (protein) AND TITLE-ABS-KEY (isolate))
Web of Science	Ultrasound (All Fields) or Ultrasonication (All Fields) or Ultrasonic (All Fields) or Sonication (All Fields) and Protein (All Fields) and isolate (All Fields) and Food Science Technology (Web of Science Categories)
Wiley	“Ultrasound OR ultrasonication OR ultrasonic OR sonication” in Title and “protein AND isolate” in Title
Consensus	PROMPT: Application of ultrasound to improve physicochemical and techno-functional properties in protein isolates from cereals and pseudocereals

Note: The search strings are reported exactly as entered and executed in the corresponding database interfaces.

**Table 2:** Research question formulated using the PICOS methodology.

PICOS Element	Description as Applied in the Review
Population	Protein isolates and equivalent isolated protein fractions derived from cereals and pseudocereals, including eligible mixed protein systems containing a cereal- or pseudocereal-derived protein as a principal component.
Intervention	Ultrasound-assisted extraction and/or post-extraction ultrasonic modification, applied alone or in combination with other processing treatments
Comparison	Untreated protein isolates, conventional extraction or processing methods, and corresponding treatments performed without ultrasound
Outcome	Physicochemical, structural, techno-functional, and digestibility-related properties
Study design	Experimental studies

**Table 3:** Methodological quality assessment and risk of bias of the articles included in the review.

Reference	Experimental Design	Control Group	Ultrasound Parameters	Statistical Analysis	Risk of Bias
Hegde et al. [26]	Factorial design of experiments; 4 treatments	Conventional protein-extraction method	200 W; 10–30 min; 5 s ON/5 s OFF	ANOVA; Tukey test (*p* < 0.05).	Low
Yang & Cheng [27]	Factorial design of experiments; sample size or number of runs not specified	None (comparative treatments only)	19.9 W; 25 min; 20 kHz	ANOVA; LSD test (*p* < 0.05).	Low
Igartúa et al. [28]	Factorial design of experiments; 10 treatments	Untreated rice protein isolate	100% amplitude; 5 min; 30 s ON/30 s OFF cycle	ANOVA with Tukey test.	Low
He et al. [29]	Factorial design of experiments; 5 treatments	None (comparative treatments only)	20 kHz; 380 W; 10 min; 5 s ON/2 s OFF	ANOVA, SPSS v19.0, *p* < 0.05.	Moderate
Xie et al. [30]	Single-factor orthogonal design; 16 runs, 4 factors	Commercial soy protein isolate (SPI)	400 W, 10 min	ANOVA (*p* < 0.05).	Moderate
Luo et al. [31]	Factorial design of experiments	Untreated quinoa protein isolate	20 kHz; 14.4 W; 5 min and 15 min; 30 s ON/30 s OFF	ANOVA (*p* < 0.05).	Low
Jin et al. [32]	Factorial design of experiments; 8 treatments	Untreated buckwheat protein isolates	20 kHz; 60% amplitude; 10 s ON/5 s OFF; 10 min	ANOVA; Tukey test (*p* < 0.05).	Low
Omura et al. [33]	Box–Behnken experimental design; 13 runs	Untreated protein isolates	20 kHz; up to 712.5 W; 45 mL sample	ANOVA; response-surface methodology (RSM).	Low
Tomé & García [34]	Factorial design of experiments; 7 treatments	Untreated amaranth protein isolates	30 kHz; 100 W; 15–30 min; 30%, 60%, 90% amplitude	ANOVA; Tukey test (*p* < 0.05).	Low
Mir, Riar & Singh [35]	Factorial design of experiments; 5 treatments	Untreated quinoa protein isolates	20 kHz; 500 W; 25% amplitude; 5–35 min	ANOVA; Duncan test (*p* ≤ 0.05).	Moderate
Zhang et al. [36]	Factorial design of experiments; 7 treatments	Untreated rice dreg protein isolates	20/40 kHz; 10 s ON/5 s OFF; 15 min; 400 W L^−1^	ANOVA; Tukey test (*p* < 0.05).	Low
Silventoinen & Sozer [37]	Factorial design of experiments; 5 treatments	Untreated barley protein isolate	20 kHz; 100% amplitude; 2.5 min, cooling, + 2.5 min	ANOVA; Tukey test (*p* < 0.05).	Low
Qin, Luo & Peng [38]	Factorial design of experiments; 12 treatments	Untreated quinoa protein isolates	100 W; 20 min; 25 °C	ANOVA (*p* < 0.05).	Moderate
Li et al. [39]	Factorial design of experiments; 4 treatments	Untreated QPI and a heat-treated sample (90 °C/30 min)	20 kHz; 200–600 W; 20 min; 79.4–158.79 W cm^−2^	ANOVA; Duncan test (*p* ≤ 0.05).	Low
Xue et al. [40]	Factorial design of experiments; 3 treatments	BPI-dextran conjugates obtained by classical heating	544.59 W m^−2^; 80 min; 70 °C	ANOVA; Duncan test (*p* ≤ 0.05).	Moderate
Qin et al. [41]	Factorial design of experiments; 5 treatments	Untreated soy protein isolate	40 kHz; 300 W; 10:1 (*w*/*w*); 0–40 min	ANOVA (*p* < 0.05), SPSS v19.0.	Low
Cen et al. [42]	Factorial comparative design; 5 ultrasound-power levels, including the control, combined with 3 vegetable-oil phases	Untreated QPI dispersion (0 W), with the corresponding oil phase	0, 150, 300, 450, and 600 W; 20 min; 25 °C; frequency and duty cycle not reported	At least 3 independent samples; ANOVA; Duncan’s multiple-range test (*p* < 0.05).	Moderate
Chen et al. [43]	Central composite design; 20 runs and 3 factors, coupled with ANN–GA optimization	QPI–GA conjugate prepared by classical wet heating (QGCH); untreated QPI–GA mixture (QG)	200–800 W; 10–60 min; 50–90 °C; 2 s ON/2 s OFF	Assays conducted at least in triplicate; one-way ANOVA (*p* < 0.05); SPSS v26.0; CCD and ANN–GA optimization.	Low
He et al. [44]	Single-factor experiments followed by a 4-factor Box–Behnken response-surface design	Traditional alkaline extraction followed by isoelectric precipitation	Time: 30–180 min; temperature: 25–75 °C; pH 8–13; solid-to-liquid ratio 1:5–1:30	Mean ± SD; one-way ANOVA and multiple comparisons using SPSS v17.0; response-surface analysis using Design-Expert v8.0.	Moderate
Li & Xiong [45]	Factorial comparative design involving ultrasound treatment and different pH conditions	Non-sonicated OPI at each corresponding pH	20 kHz; approximately 80 W; 70% amplitude; 5 min; 25 mL sample; pH 7 during treatment	3 independent trials, each with triplicate analyses; ANOVA; Tukey’s HSD test (*p* < 0.05).	Low
Li et al. [46]	Two-factor design: ultrasound treatment × cysteine concentration; 2 ultrasound conditions and 5 cysteine levels	Untreated OPI containing 0 mg cysteine g^−1^ protein; matched non-sonicated OPI at each cysteine concentration	20 kHz; 60 W; 50% amplitude; 15 min; 30 s ON/30 s OFF, with 2.5 min intervals; 20 °C	NMR analyses performed in duplicate; other experiments in triplicate; ANOVA; Tukey’s HSD test (*p* < 0.05).	Low
Li et al. [47]	Factorial design: 3 sonication conditions × 5 homogenization-pressure levels; 15 treatment combinations	Untreated OPI without ultrasound or high-pressure treatment; HIU-only and HP-only groups were also included	20 kHz; 60 W; 50% amplitude; 15 min; 30 s ON/30 s OFF, with 2.5 min intervals; treatment at 20 or 60 °C	ANOVA; Tukey’s HSD test (*p* < 0.05).	Low
Rafique et al. [48]	Completely randomized single-factor design; 4 ultrasound-power treatments	Untreated oat protein isolate	0, 200, 400, and 600 W; 25 kHz; 15 min; 30 °C; OPI dispersion at 5% (*w*/*v*)	Triplicate experiments; ANOVA under a completely randomized design; Tukey’s HSD test (*p* < 0.05).	Moderate
Wang et al. [49]	Factorial design: 4 pH conditions × 3 processing strategies—pH shifting alone, pH shifting plus heat, and pH shifting plus sonication	Untreated QPI dispersion at pH 7; corresponding pH-shift-only and heat-treated groups	20 kHz; actual acoustic power 12.5 W; 30 min; 30 s ON/30 s OFF; ice-water bath; pH 2, 7, 10, and 12	Mean ± SD; ANOVA followed by LSD test; Pearson correlation analysis; SPSS v21.0; *p* < 0.05.	Low
Yang et al. [50]	Comparative multi-treatment design; native QPI, thermally acid-hydrolyzed QPI, enzymatically hydrolyzed QPI, and ultrasonicated QPI	Native, unmodified quinoa protein isolate	300 W; 20 min; QPI-to-water ratio 1:20 (*w*/*w*)	Mean ± SD; ANOVA followed by LSD post hoc test; SPSS Statistics v25; *p* < 0.05.	Moderate
Zuo et al. [51]	Single-factor ultrasound-energy-density design; 4 acoustic-energy levels followed by structural, interfacial, thermal analyses	Native QPI treated	20 kHz; 360 W; 0, 1, 5, and 10 kJ mL^−1^; probe sonication under an ice bath	Mean ± SD; ANOVA; Duncan’s multiple-range test (*p* < 0.05).	Low

**Table 4:** Physicochemical, structural, techno-functional, and digestibility-related properties evaluated in the included studies.

Treatment	Protein Source	S	EAI/ESI	FC/FS	WHC/OHC	IVPD	Particle Size	Str 2°/3°
Cereals								
US + HHP [26]	Pearl millet (80–81%)	x	x	x	x	x	NC	x
pH-shift + US + heat [28]	Rice (83.6%)	x	x	NA	NA	NA	x	NC
US [30]	Wheat germ (90.9% ± 1.7%)	NC	x	x	x	NA	NA	NR
US [33]	Rice (80–83%)	NC	x	x	x	NA	x	NR
Dual-frequency US [36]	Rice residue (89–93%)	NC	NA	NA	NA	NA	x	x
US + pH-shift [37]	Barley (85.9 ± 0.4%)	x	NA	NA	NA	NA	x	NC
US + transglutaminase [41]	Wheat gluten (≥90%)	NA	NA	NA	x	NA	x	x
HIUS [45]	Oat (83%)	x	NA	NA	NA	NA	x	NC
HIUS + disulfide cleavage [46]	Oat (91.8%)	NA	NA	NA	x	NA	x	NC
HIUS + HP [47]	Oat (95.3%)	x	x	NA	NA	NA	x	NC
US [48]	Oat *	NA	NA	NA	NA	NA	NA	x
Pseudocereals								
US [27]	Quinoa (91.6%)	NA	NA	NA	NA	NA	x	NA
US [29]	Quinoa (80%)	NA	NA	NA	NA	x	NC	NC
US + HHP [31]	Quinoa (~90%)	x	NA	NA	NA	NA	x	NC
US [32]	Buckwheat *	NA	NA	NA	NA	x	x	x
US [34]	Amaranth (84 ± 2%)	x	x	NA	NA	NA	x	NC
HIUS [35]	Quinoa (84.5 ± 0.5%)	NC	x	NA	x	NA	NC	NC
US [38]	Quinoa (~80%)	NA	x	NA	NA	NA	x	NC
US [39]	Quinoa *	x	NA	NA	x	x	NA	x
HIUS + glycation [40]	Buckwheat *	x	x	NA	NA	NA	x	x
US [42]	Quinoa (≥90%)	NA	x	NA	NA	NA	x	NA
US-assisted wet heating (glycation) [43]	Quinoa *	x	x	NA	NA	NA	x	NC
US + heat [44]	Quinoa (87.2%)	NC	NA	NA	NA	x	NA	NA
pH-shift + US or heat [49]	Quinoa (91.6%)	x	x	x	NA	NA	x	x
US [50]	Quinoa *	x	NC	NA	NA	NA	x	NA
US [51]	Quinoa (86.7%)	NA	NA	NA	NA	NA	x	x

* Protein content was not reported in the original study. Note: “x” indicates that the corresponding property was evaluated and reported in the original study. NA, not assessed; NR, not reported; NC, not classifiable. These indicators describe outcome coverage only and do not represent the direction or magnitude of the ultrasound-induced effect.

## Data Availability

The data supporting this systematic review are available in Zenodo at https://doi.org/10.5281/zenodo.16928429 (accessed on 18 September 2026). The repository contains the records retrieved from Scopus, Web of Science, Wiley, and Consensus, corresponding to the raw search results and the initial screening dataset. Access to restricted files may be granted by the corresponding author upon reasonable request for academic and research purposes. The extracted data and author-derived synthesis supporting the findings of this review are presented in the article and its Supplementary Materials.

## Supplementary Materials

The following supporting information can be downloaded at: https://www.mdpi.com/article/10.3390/foods15193498/s1, File S1: Completed PRISMA 2020 checklist. File S2: Risk-of-bias assessment. Table S1 [Domain-based criteria adapted from ROBINS-I principles and decision rules for laboratory experimental studies in food science]; Table S2 [Semiquantitative domain-level risk-of-bias assessment of the 26 included studies]; Table S3 [Interpretation of the overall risk-of-bias score].

## Abbreviations

The following abbreviations are used in this manuscript:

AI	Artificial intelligence
ANOVA	Analysis of variance
EAI	Emulsifying activity index
EGCG	Epigallocatechin-3-gallate
ESI	Emulsifying stability index
FC	Foaming capacity
FS	Foaming stability
HHP	High hydrostatic pressure
HIUS	High-intensity ultrasound
IVPD	In vitro protein digestibility
LCA	Life cycle assessment
OHC	Oil-holding capacity
PICOS	Population, Intervention, Comparison, Outcomes, and Study Design
PLC	Programmable logic controller
PRISMA	Preferred Reporting Items for Systematic Reviews and Meta-Analyses
ROBINS-I	Risk of Bias in Non-randomized Studies of Interventions
RSM	Response surface methodology
SPI	Soy protein isolate
US	Ultrasound
WHC	Water-holding capacity
pI	Isoelectric point
