# Supplementary material for: Application of Ultrasound for the Modification of Techno-Functional and Physicochemical Properties in Cereal and Pseudocereal Protein Isolates: A Systematic Review
Source: Foods. 2026 Sep 29;15(19):3498. doi: 10.3390/foods15193498 (PMC13665040; doi:10.3390/foods15193498)
Supplement: Supplementary file 1 [file foods-15-03498-s001.zip › S2 - Supplementary_Risk_of_Bias - FOODS.pdf]

Risk-of-Bias Assessment

Methodological note. A domain-based methodological appraisal adapted from the principles of ROBINS-I (Risk Of Bias In Non-randomized Studies of Interventions) was developed for laboratory experimental studies in food science. The seven domains were operationalized for studies involving ultrasound-treated cereal and pseudocereal protein matrices. Each domain was scored as low risk (2 points), moderate risk (1 point), or high risk (0 points), and the domain scores were summed to obtain a maximum of 14 points. This semiquantitative scoring system was developed specifically for the present systematic review and should not be interpreted as the original ROBINS-I scoring system.

Table S1. Domain-based criteria adapted from ROBINS-I principles and decision rules for laboratory experimental studies in food science.

Domain	Adapted criterion	Indicators evaluated	Low risk (2)	Moderate risk (1)	High risk (0)
D1. Bias due to confounding variables	Evaluates whether pre-existing or deliberately different conditions between treatment and comparator groups could explain the observed effect independently of ultrasound.	pH, protein concentration, solvent/ionic conditions, matrix composition, pretreatments, comparator equivalence, and other between-group factors. Unplanned heating generated during sonication is evaluated primarily in D4.	Relevant between-group conditions were controlled, matched, or intentionally incorporated into the design so that the ultrasound contribution remained interpretable.	One or more relevant between-group conditions were incompletely controlled or described, but the ultrasound contribution remained reasonably interpretable.	Major uncontrolled between-group differences prevented reasonable attribution of the observed effect to ultrasound.
D2. Bias in selection and preparation of experimental samples	Examines whether the experimental samples were sufficiently homogeneous and comparable before the intervention.	Sample origin, isolate purity/source, extraction or preparation procedure, pretreatments, batch comparability, and consistency of sample handling before treatment.	Sample origin and preparation were clearly described and treatment/comparator groups were derived from comparable material.	Sample comparability was generally acceptable, but relevant information on origin, purity, batch, pretreatment, or preparation was incomplete.	Sample selection or preparation differed substantially between groups, or comparability could not be established.
D3. Bias in classification and description of the intervention	Determines whether the ultrasonic treatment and comparator were described with sufficient precision to identify the intervention and interpret the effective acoustic exposure.	Frequency, power and/or amplitude, treatment time, duty cycle, temperature target, equipment/probe information, treated volume/concentration, and comparator definition.	The principal ultrasound parameters and comparator were sufficiently described to characterize the intervention with high reproducibility.	One relevant parameter was absent or incompletely described, but the intervention remained identifiable and interpretable.	Several key parameters were absent, or the equipment/exposure could not be adequately characterized or reproduced.
D4. Bias due to deviations from the intended treatment	Evaluates whether the applied treatment may have deviated materially from its intended conditions during sonication.	Temperature control or monitoring, cooling strategy, treatment geometry, probe immersion/position where relevant, and other measures used to control unintended process deviations.	Critical treatment conditions were actively controlled or monitored and no relevant deviation was evident.	Some control or mitigation was reported, but achieved conditions were incompletely verified or one relevant control detail was missing.	No meaningful control or monitoring of a critical deviation was reported, or a substantial unintended deviation was documented.
D5. Bias due to missing or incomplete data	Assesses whether incomplete outcome data or insufficient replication/dispersion reporting could affect interpretation.	Number of replicates, numerical completeness, mean values, SD/SE or other dispersion measures, missing values, and completeness of quantitative reporting.	Relevant outcomes were quantitatively reported with adequate replication and dispersion measures.	Some replication, numerical values, or dispersion measures were incomplete, but the main findings remained interpretable.	Essential outcome data or replication information were missing to an extent that compromised reliable interpretation.
D6. Bias in measurement of outcomes	Evaluates the validity, reliability, and consistent application of the methods used to measure physicochemical, structural, and techno-functional outcomes.	Use of recognized or validated analytical methods, sufficient methodological description, and consistent measurement across groups.	Recognized or adequately described methods were applied consistently to the relevant groups.	Methods were generally appropriate but one or more relevant validation, implementation, or quantification details were incomplete.	Measurement methods were inappropriate, insufficiently described, or inconsistently applied, substantially compromising reliability.
D7. Bias in selection of the reported result	Evaluates whether the results reported correspond to the outcomes described in the methods and whether favorable, null, or unfavorable findings appear to have been selectively presented.	Correspondence between methods and results, completeness of reported outcomes, and evidence of selective omission or emphasis.	Reported outcomes were consistent with the methods and there was no evident selective reporting.	Minor reporting discrepancies or incomplete presentation were present but were unlikely to materially alter interpretation.	Important omissions or selective reporting were evident and could distort interpretation of the treatment effects.

Scoring rule: Low risk = 2 points; moderate risk = 1 point; high risk = 0 points per domain. Maximum total score = 14 points.

Table S2. Semiquantitative domain-level risk-of-bias assessment of the 26 included studies.

Reference	D1	D2	D3	D4	D5	D6	D7	Total	Overall risk
Cen et al. [42]	2	1	1	1	2	2	2	11	Moderate
Chen et al. [43]	2	1	2	1	2	2	2	12	Low
He et al. [44]	1	1	0	1	2	2	2	9	Moderate
He et al. [29]	2	2	1	1	1	2	2	11	Moderate
Hegde et al. [26]	2	2	1	2	2	2	2	13	Low
Igartúa et al. [28]	2	2	1	1	2	2	2	12	Low
Jin et al. [32]	2	2	2	2	2	2	2	14	Low
Li & Xiong [45]	2	2	2	1	2	2	2	13	Low
Li et al. [39]	1	2	2	1	2	2	2	12	Low
Li et al. [46]	2	2	2	2	1	2	2	13	Low
Li et al. [47]	2	2	2	2	1	2	2	13	Low
Luo et al. [31]	2	2	2	2	1	2	2	13	Low
Mir, Riar & Singh [35]	1	2	1	0	2	2	2	10	Moderate
Omura et al. [33]	2	2	2	1	1	2	2	12	Low
Qin et al. [41]	2	2	2	2	2	2	2	14	Low
Qin, Luo & Peng [38]	1	1	0	0	2	2	2	8	Moderate
Rafique et al. [48]	1	1	2	2	1	1	2	10	Moderate
Silventoinen & Sozer [37]	2	2	2	2	2	1	2	13	Low
Tomé & García [34]	2	2	2	2	2	2	2	14	Low
Wang et al. [49]	2	2	2	2	2	2	2	14	Low
Xie et al. [30]	1	1	0	0	2	2	2	8	Moderate
Xue et al. [40]	1	2	1	1	1	2	2	10	Moderate
Yang & Cheng [27]	2	2	1	1	2	2	2	12	Low
Yang et al. [50]	1	1	1	0	1	2	2	8	Moderate
Zhang et al. [36]	2	2	2	2	1	2	2	13	Low
Zuo et al. [51]	2	2	2	1	2	2	2	13	Low

Table S3. Interpretation of the overall risk-of-bias score.

Total score	Overall risk of bias	Interpretation
12–14	Low	High methodological rigor and adequate experimental control; identified limitations are minor and unlikely to materially alter interpretation.
8–11	Moderate	One or more methodological or reporting limitations are present, but the study remains sufficiently interpretable and the limitations do not invalidate the overall findings.
0–7	High	Important limitations across several domains may compromise attribution or interpretation of the observed treatment effects.

Summary of overall judgments. Among the 26 included studies, 17 were classified as having low overall risk of bias, 9 as moderate risk, and none as high risk.
